# Comparison of pectoral angina misconception among Iranian nurses, nursing students and patients

**Published:** 2015

**Authors:** A Mohajjel Aghdam, H Hasankhani, Z Shamshiri, S Ghaffari

**Affiliations:** * Tabriz University of Medical Sciences-mohajjela@tbzmed.ac.ir

**Keywords:** misconception, nurse, nursing student, angina pectoris

## Abstract

**Background and objective:** previous studies revealed some angina misconception among patients and health care providers. The aim of this study was to assess the misconceptions about angina held by nurses, nursing students and patients.

**Materials and methods:** In this cross sectional study, 120 nurses, 120 nursing students, and 120 patients with angina pectoris in Iran participated. Data were gathered by using the York angina belief Questionnaire version 1. The mean of angina misconception were compared by using ANOVA analysis of variance. The correlations between the questionnaire and the variables were calculated by regression. α < 0.05 was considered significant.

**Results:** Nursing students had a significantly lower misconception than patients and nurses (39.03 ± 6.35 vs. 43.70 ± 7.22 in nurses and 43.78 ± 5.77 in patients, P = 0.001). However, the differences between nurses and patients with angina, regarding the misconception score, were not significant: 43.70 ± 7.22 vs. 43.78 ± 5.77, P = 0.9, and no statically significant association was made between age, sex, education, training and number of misconception in patients, nurses and nursing students.

**Conclusion:** Nurses have the most pregnant relationship with patients at different stages of their treatment and can play an important role in assessing their misconceptions and intervention to dispel them. It seems that the nursing students and the nurses’ continual professional educations should be emphasized to use the scientific knowledge to dispel the misconceptions in patients.

## Introduction

According to the National Center for Health Statistics 2011 report, cardiovascular disease (CVD) remains the leading cause of mortality in the United States in men and women of every major ethnic group. It accounted for nearly 616,000 deaths in 2008 and was responsible for 1 in 4 deaths in the U.S. in the same year. CAD is the most common type of heart disease and, in 2008, 405,309 individuals died in the U.S. from this specific etiology. Every year, approximately 785,000 Americans suffer a first heart attack and another 470,000 will suffer an additional myocardial infarction (MI). In 2010, CAD alone was projected to cost the U.S. $108.9 billion, including the cost of health care services, medications, and lost productivity. Recent data indicate that the Iranian adult population has a high prevalence of CAD risk factors [**1**]. The high prevalence and morbidity associated with CAD in Iran is one of the most pressing health problems [**2**].

Studies have indicated that the improvement of knowledge regarding the cardiac disease and healthy behaviors such as prevention of unhealthy diet, smoking and alcohol drinking have decreased the rate of mortality and morbidity of patients [**3**-**5**]. However, the perception of health care providers and patients regarding these behaviors is not consistent due to the different sources of knowledge that they give [**6**]. The misconception leads to several problems for patients, family, and health care system, because of a more mortality and morbidity rate, slow recovery and increasing duration of hospitalization [**7**]. Moreover, sometimes the cardiac misconception may occur in patients due to the lack of a correct information of health care providers [**8**,**9**]. Previous reports revealed that misconception decreasing, leads to the improvement of functions in patients with cardiac disease [**10**]. Therefore, in some countries such as Britain, the management of misconception in patients and health care providers is a part of the cardiac rehabilitation [**11**]. In Iran, studies evaluating the cardiac misconception among patients and health care providers are rare. Hence, in this comparative study, the level of angina misconception and demographic data that affect the level of misconception among patients, nurses, and students of nursing was assessed by using the York questionnaire.

## Materials and methods 

In this comparative study, 120 patients, 120 nurses, and 120 students of nursing from the teaching hospitals of Tabriz University of Medical Sciences were recruited. The inclusion criteria for the patients were hospitalization due to angina pectoris, ability to verbal communication, written consent, age ≥ 18 years and stable hemodynamics. The exclusion criteria for patients were heart failure and mental disease. The criteria for the enrollment of nurses were at least six months of clinical work experience, no diagnosis of angina, Bachelor Degree of Nursing, or higher levels. Moreover, the nursing students were selected from the students in the Nursing and Midwifery Faculty of Tabriz University of Medical Sciences who passed the cardiovascular course.

**Ethical consideration**

**The study protocol was approved by the ethical committee of Tabriz University of Medical Sciences.**

**The questionnaire**

To assess the angina misconception, York questionnaire version 1 (YCBQ) was used, that is used to assess the concept of angina. It included 16 questions about angina, each question scored from 0 (Strongly disagree) to 4 (strongly agree) with “I don’t have any idea about this” scoring 2). Higher scores reflected a stronger misconception. The questionnaire and the aim of study were explained to patients, then the face-to-face interview was performed and the questionnaire was completed. Nursing students and nurses were also informed about the study procedure and they completed the questionnaires.

**Statistical analyses**

Data were analyzed by using SPSS version 20. Categorical data were presented as numbers (%), and the continuous data as mean ± SD. The distribution of the data in three groups was normal, so, the mean of angina misconception was compared by using the ANOVA analysis of variance. The correlations between the misconception and the demographic data were calculated by regression. α < 0.05 was considered significant.

## Results

120 nursing students, with a mean age of 21 years (43 men, 77 women), 120 nurses with a mean age of 27 years (8 men, 112 women) and 120 patients with a mean age of 65 years (70 men, 50 women), were analyzed. The mean of misconception in nursing students was significantly lower than in nursing and patients (39.03 ± 6.35 vs. 43.70 ± 7.22 in nurses and 43.78 ± 5.77 in patients, P = 0.001). However, the difference between the patients and the nurses was not significant (43.70 ± 7.22 vs. 43.78 ± 5.77, P = 0.9) (**Table 1**). 

**Table 1:** The misconception score in patients, nurses and students

Groups	mean	SD	P
Patients	43.78	5.77	0.64
Students	39.03	6.35	
Nurses	43.70	7.22	

Moreover, the correlation between age, sex, education, and training was evaluated and it was indicated that these factors were not correlated with the misconception score in patients, nurses, and nursing students (**Table 2**-**4**).

**Table 2:** Correlation between sex, age and education level with the misconception score in patients

Patients		Mean ± SD	P
sex	Male	42.8 ± 5.8	0.80
	Female	45.1 ± 5.4	
age	<60	44.1 ± 5.5	0.69
	>60	44.01 ± 5.3	
education	illiterate	45.11 ± 5.8	0.48
	<high school	42.06 ± 5.03	
	university	42.3 ± 5.06	

**Table 3:** Correlation between sex, age and education level with the misconception score in students

student		Mean ± SD	P
sex	Male	38.7 ± 6.7	0.21
	Female	39.2 ± 6.1	
age	20-24	38.8 ± 6.5	0.60
	>24	38.7 ± 6.6	
Education	1	40.2 ± 5.5	0.23
	2	39.1 ± 6.9	
	3	36.3 ± 6.3	
	4	39.1 ± 6.2	

**Table 4:** Correlation between sex, age and education level with the misconception score in nurses

Nurses		Mean ± SD	P
sex	Male	42.3 ± 9.3	0.20
	Female	43.8 ± 7	
age	20-30	43.7 ± 7.11	0.23
	>30	44.5 ± 8.2	
Education	College	47.00 ± 00	0.11
	Bachelor	43.8 ± 7.2	
	Master	36.5 ± 3.5	
Work experience	<1 year	48.3 ± 5.9	0.54
	1-5	46.4 ± 7.2	
	5-10	42. ± 6.2	
	>10	42.9 ± 7.5	

## Discussion 

In this study, the misconception among patients and nurses was significantly higher than in nursing students. In line with our findings, a study by Lin et al. in 2008 showed similar results and indicated that nurses held a higher misconception than the nurse students [**12**]. Consistently, in 2010, another study was performed in America, in which Kandula indicated that 89% of the participants declared that their information about the heart disease and its risk factors are low. Moreover, among them, only about 10% controlled the cardiovascular disease risk factors such as blood pressure and lipid profile. Additionally, 53% of them believed that the cardiac disease is not avoidable [**13**]. Previous reports have indicated that several factors impact on the level of cardiac misconception such as training and education. For instance, a study by Angus et al. in 2012 revealed that education was significantly correlated with the level of misconception in health care providers. They showed that staff without training had a higher rate of cardiac misconception [**14**]. However, in contrast to Angus et al. findings, in the present experience, the level of cardiac misconception was not correlated to the education level in patients, students, and nurses. Moreover, Angus et al. highlighted that the staff with more work experience had a lower cardiac misconception [**14**], but, as opposed to these results, in our practice, the work experience was not correlated with the cardiac misconception score. In current practice, we indicated that the cardiac misconception was not correlated with sex and age. In line with our finding, Jensen et al. indicated in a study performed in 2008 that the difference between males and females regarding cardiac misconception was not significant [**15**]. Moreover, no correlation between the passed trimesters by the nursing students and cardiac misconception was revealed in the current study, but in contrast to our results, a study by Shaw et al. indicated that students in trimesters six had less misconception than students in trimesters 1 and four [**16**].

Other studies in this field presupposed that the cultural differences have an important role in coronary disease misconception. To evaluate the hypothesis, Lin et al. compared the British and Taiwanese patients with the cardiac disease ones in a study and indicated that the Taiwanese patients significantly had more cardiac misconceptions than British patients. They concluded that this difference might be related to different health information and health care facilities in the two countries [**17**].

This was a cross sectional study that limited our ability to evaluate the cause of angina misconception among three groups. Moreover, the selected patients were from the teaching hospitals of Tabriz University of Medical Sciences that limited our ability to generalize the results of this study to the hospitals across Iran. Further multicentric studies across Iran are required to confirm the results reported here and to evaluate the incorrect believes regarding the nature of angina among patients, general population, and health care providers.

## Conclusion

Nurses should be able to assess their patients’ misconception to dispel them. Our study showed that there are no significant differences between the nurses and the patients regarding the rate of angina misconception. Therefore, nurses cannot dispel misconceptions from their patients. Our finding provides information for nursing practice and education. More multicentric studies with large sample sizes are required to confirm the results reported here.

**Acknowledgements**

We would like to thank the nurses, nursing students and patients who participated in this study.

The authors declare no conflict of interest. 
